# *β-D-XYLOSIDASE 4* modulates systemic immune signaling in *Arabidopsis thaliana*

**DOI:** 10.3389/fpls.2022.1096800

**Published:** 2023-02-02

**Authors:** Kornelia Bauer, Shahran Nayem, Martin Lehmann, Marion Wenig, Lin-Jie Shu, Stefanie Ranf, Peter Geigenberger, A. Corina Vlot

**Affiliations:** ^1^ Department of Environmental Science, Institute of Biochemical Plant Pathology, Helmholtz Munich, Neuherberg, Germany; ^2^ Faculty of Biology, Ludwig-Maximilians University of Munich, Munich, Germany; ^3^ TUM School of Life Sciences Weihenstephan, Chair of Phytopathology, Technical University of Munich, Freising, Germany; ^4^ Department of Biology, University of Fribourg, Fribourg, Switzerland; ^5^ Faculty of Life Sciences: Food, Nutrition, and Health, Chair of Crop Plant Genetics, University of Bayreuth, Kulmbach, Germany

**Keywords:** plant immunity, plant defense, systemic acquired resistance, cell wall, xylosidase, xylose, fucose, putrescine

## Abstract

Pectin- and hemicellulose-associated structures of plant cell walls participate in defense responses against pathogens of different parasitic lifestyles. The resulting immune responses incorporate phytohormone signaling components associated with salicylic acid (SA) and jasmonic acid (JA). SA plays a pivotal role in systemic acquired resistance (SAR), a form of induced resistance that - after a local immune stimulus - confers long-lasting, systemic protection against a broad range of biotrophic invaders. β-D-XYLOSIDASE 4 (BXL4) protein accumulation is enhanced in the apoplast of plants undergoing SAR. Here, two independent *Arabidopsis thaliana* mutants of *BXL4* displayed compromised systemic defenses, while local resistance responses to *Pseudomonas syringae* remained largely intact. Because both phloem-mediated and airborne systemic signaling were abrogated in the mutants, the data suggest that BXL4 is a central component in SAR signaling mechanisms. Exogenous xylose, a possible product of BXL4 enzymatic activity in plant cell walls, enhanced systemic defenses. However, GC-MS analysis of SAR-activated plants revealed BXL4-associated changes in the accumulation of certain amino acids and soluble sugars, but not xylose. In contrast, the data suggest a possible role of pectin-associated fucose as well as of the polyamine putrescine as regulatory components of SAR. This is the first evidence of a central role of cell wall metabolic changes in systemic immunity. Additionally, the data reveal a so far unrecognized complexity in the regulation of SAR, which might allow the design of (crop) plant protection measures including SAR-associated cell wall components.

## Introduction

Diverse environmental stimuli challenge plants to continuously adapt to their surroundings. Host-invading pathogens and other stress-associated stimuli prompt plants to drive up inducible immune responses. The associated intricate metabolic signaling network affects stress responses and defense against an array of biotic invaders (Spoel and Dong, 2012).

In response to infection, plants activate a number of defense responses. These include pathogen-associated molecular pattern (PAMP)-triggered immunity (PTI) after the perception of virulent invaders, and effector-triggered immunity (ETI) upon detection of avirulent attackers (Jones and Dangl, 2006; Spoel and Dong, 2012). PTI is an important component of basal immunity against host-adapted pathogens. It depends on the recognition of elicitors (PAMPs) on the pathogen surface by plant cell surface-localized pattern recognition receptors (PRRs), and impedes pathogen propagation (Jones and Dangl, 2006). ETI is initiated by intracellular nucleotide-binding, leucine-rich repeat receptors (NLRs) after sensing of pathogen-derived effector molecules (Cui et al., 2015). Activation of ETI provides an effective and long-lasting resistance to a broad range of pathogens. Both PTI and ETI rely on the phenolic phytohormone salicylic acid (SA) and promote defense against (hemi-)biotrophic pathogens (Spoel and Dong, 2012; Cui et al., 2015; Ngou et al., 2022). Proper ETI defense induction relies on functional PTI recognition and signaling, while PTI is boosted by ETI signaling components (Yuan et al., 2021; Ngou et al., 2022). Therefore, the induction of SA-related defense gene expression as well as the accumulation of immune-modulating components, such as reactive oxygen species (ROS), appears to be the result of a synergistic interplay between PTI and ETI (Yuan et al., 2021; Ngou et al., 2022).

Both PTI and ETI trigger the establishment of inducible defense responses in distal, healthy leaves of locally infected plants (Spoel and Dong, 2012; Vlot et al., 2021). This type of induced defense is known as systemic acquired resistance (SAR) and is associated with interconnected stress-activated processes and long-distance signaling *via* both vascular and airborne routes (Vlot et al., 2021). A local treatment of *Arabidopsis thaliana* (*A. thaliana*) with PTI- or ETI-inducing bacteria, for example, initiates SAR protecting systemic, healthy tissues from a secondary infection with virulent pathogens (Mishina and Zeier, 2007; Shah and Zeier, 2013). The establishment of an effective SAR response depends on two interconnected and synergistically regulated pathways that are respectively associated with SA and pipecolic acid (Pip) (Gao et al., 2015; Vlot et al., 2021). First, SA functions during SAR in a positive feedback loop with the key regulator ENHANCED DISEASE SUSCEPTIBILITY 1 (EDS1), which is necessary for both local SAR signal generation/transmission and systemic signal perception/propagation (Breitenbach et al., 2014; Cui et al., 2017; Vlot et al., 2021). SA-related signals commonly promote transcript accumulation of *PATHOGENESIS-RELATED* (*PR*) genes, including the SA and SAR marker gene *PR1* (Van Loon et al., 2006). Second, the non-protein amino acid Pip and its presumed bioactive conversion product N-hydroxy-pipecolic acid (NHP) promote SAR in association with the NHP biosynthetic enzyme FLAVIN-DEPENDENT MONOOXYGENASE1 (FMO1) (Navarova et al., 2012; Chen et al., 2018; Hartmann et al., 2018; Wang et al., 2018a). Long distance signaling *via* the phloem has been associated with SA, its methylated derivative methyl salicylate, and also with Pip/NHP promoting SAR in a positive feedback loop with glycerol-3-phosphate (G3P) and the predicted lipid transfer protein AZELAIC ACID INDUCED 1 (AZI1), which in turn cooperates with further predicted lipid transfer proteins (EARLY ARABIDOPSIS ALUMINUM INDUCED 1, DEFECTIVE IN INDUCED RESISTANCE 1 (DIR1) and DIR1-like) (Maldonado et al., 2002; Park et al., 2007; Jung et al., 2009; Chanda et al., 2011; Champigny et al., 2013; Cecchini et al., 2015; Chen et al., 2018; Wang et al., 2018a; Wang et al., 2018b; Hartmann and Zeier, 2019; Lim et al., 2020; Holmes et al., 2021). Additionally, SAR is associated with airborne molecules that contribute to long-distance signaling within and between plants (Riedlmeier et al., 2017; Wenig et al., 2019; Brambilla et al., 2022). Airborne signals that are recognized as defense cues, inducing SAR-like defense in neighboring receiver plants, include the monoterpenes α/β-pinene and camphene (Riedlmeier et al., 2017). Comparable responses have been observed in *A. thaliana* and in the crop plants lima bean (*Phaseolus lunatus*) and barley (*Hordeum vulgare*) in response to terpenoids (*A. thaliana*), the fatty acid-derived volatile nonanal (lima bean and barley), and the apocarotenoid signal β-ionone (barley) (Yi et al., 2009; Frank et al., 2021; Brambilla et al., 2022). Systemically, the SAR-associated protein LEGUME LECTIN-LIKE PROTEIN 1 (LLP1) is essential for recognition or transduction of both phloem-associated and airborne signals for the establishment of SAR (Breitenbach et al., 2014; Wenig et al., 2019). This function of LLP1 is further supported by its enhanced transcript accumulation in systemic tissues during SAR (Breitenbach et al., 2014).

The extracellular space which includes the apoplast and the plant cell wall, is a prominent area where host-pathogen interactions occur (Doehlemann and Hemetsberger, 2013; Malinovsky et al., 2014). This area, for example, constitutes the space where PRR-mediated recognition of PAMPs takes place (Saijo et al., 2018; Schellenberger et al., 2019). Consequently, the apoplast and cell wall play an important role in monitoring and integrating external stimuli that activate downstream signaling. Also, modifications in the composition of cell walls are anticipated to strongly affect immunity, fitness, and developmental adaptation in plants (Malinovsky et al., 2014; Vaahtera et al., 2019; Molina et al., 2021). Specifically, pectin- and hemicellulose-associated structures participate in defense, incorporating responses *via* the hormonal signaling routes of SA, jasmonic acid (JA), and ethylene (ET) (Bethke et al., 2016; Claverie et al., 2018; Zang et al., 2019; Molina et al., 2021). Particularly, the enhanced abundance of specific xyloglucans, xylose derivatives, galactomannans, and rhamnogalacturonan-I (RG-I) molecules positively correlates with plant disease resistance.

Breitenbach et al. (2014) previously identified apoplastic proteins associated with a SAR-inducing infection of *A. thaliana*. These proteins included LLP1, which contains a predicted carbohydrate-binding (lectin) domain, and the cell wall-modifying enzyme β-D-XYLOSIDASE 4 (At5g64570, BXL4, also referred to as XYL4). BXL4 enzymatic function was described to induce the release of D-xylose from hemicellulose structures, including xylotetraose, xylobiose, xylan (of oat spelt), arabinoxylan (of rye), and (oligo)arabinoxylan (of wheat) (Minic et al., 2004). This strongly suggests that BXL4 acts as a β-D-xylosidase (Minic et al., 2004). Here, we hypothesize that BXL4 and associated cell wall-derived carbohydrates contribute to SAR. A combination of infection, petiole exudate, and plant-to-plant interaction assays reveals a new, specific role of BXL4 and associated cell wall dynamics in induced resistance. This first demonstration of cell wall function in SAR opens up possibilities to exploit natural cell wall sugars for improved (crop) plant protection.

## Materials and methods

### Plant material and growth condition

*A. thaliana* ecotype Columbia-0 (Col-0) was used throughout this work. Mutants *eds1-2*, *llp1-1*, and *bxl4-1* were previously described (Bartsch et al., 2006; Breitenbach et al., 2014; Guzha et al., 2022). The mutant line *bxl4-3* (SALK_048903) was obtained from the Nottingham Arabidopsis Stock Centre (Scholl et al., 2000). Seeds were propagated and tested for homozygosity (O’malley et al., 2015). To this end, the T-DNA insertion was confirmed in genomic DNA isolated from pooled leaf samples of at least five individual plants using primers as listed in **Supplementary Table S1**. Seeds from pooled plants that were homozygous for the T-DNA insertion were used to rear the plants for all experiments.

For experiments, plants were grown on a mixture of non-fertilized potting soil and silica sand (ratio 5:1). The plants were kept at 22°C in 10 h days with a light intensity of 100 μmol m^−2^ s^−1^ of photosynthetically active photon flux density, and at 18°C for 14 h nights. The relative humidity was kept at ~70%. 4-5 week old plants were used for all experiments.

### Pathogens and preparation of bacterial inoculum

*Pseudomonas syringae* pathovar *tomato* DC3000 (*Pst*) and *Pst* carrying the effector *AvrRpm1* (*Pst/AvrRpm1*) were used to infect plants (Breitenbach et al., 2014). Bacteria were grown at 28°C on NYGA medium (0.5% bacto proteose peptone, 0.3% yeast extract, 2% (v:v) glycerol, pH 7.0, 1.8% agar-agar; Roth) supplemented with 50 μg mL^-1^ kanamycin and 50 μg mL^-1^ rifampicin (Roth). Freshly prepared overnight cultures were used for infection assays, for which the bacteria were suspended in 10 mM MgCl_2_ (Wenig et al., 2019). The bacterial density of the suspension was determined by measuring the OD_600_ of the suspension (or a dilution thereof) on a spectrophotometer (FoodALYT bio) and by using the formula OD_600_ = 1.0 equals 10^8^ colony forming units (cfu) per mL.

### Bacterial infections

To investigate bacterial densities in infected leaves, bacterial growth curve assays were performed with *Pst* and *Pst*/*AvrRpm1*. Two fully expanded leaves per plant were either syringe-infiltrated from the lower (abaxial) side with 10^5^ cfu mL^-1^ of bacteria in 10 mM MgCl_2_ (Wenig et al., 2019). Alternatively, whole plants were sprayed from the top with 10^8^ cfu mL^-1^ of *Pst* or *Pst*/*AvrRpm1* diluted in 10 mM MgCl_2_ containing 0.01% (v:v) Tween-20 (Calbiochem, Bioscience). Similarly, corresponding mock solutions were applied by leaf infiltration of 10 mM MgCl_2_ or by spray application of 10 mM MgCl_2_ containing 0.01% (v:v) Tween-20. Infected leaves were harvested and analyzed for *in planta* bacterial titers or transcript accumulation at different time points as indicated. The *in planta* bacterial titers were determined as described (Wenig et al., 2019). In short, bacteria were extracted from three leaf discs per sample in 500 µL of 10 mM MgCl_2_, including 0.01% (v:v) Vac-In-Stuff (Silwet L-77, Lehle Seeds), while shaking (600 rotations per minute (rpm), 26°C). One hour later, samples were serially diluted and 20 µL per dilution were spotted on NYGA medium. Bacterial colonies were grown for two days at room temperature, and subsequently counted and converted to cfu per cm² of leaf tissue. Individual experiments included at least three independent samples per genotype and treatment. Biologically independent datasets were obtained from different experiments, for which seeds were sown independently.

To monitor systemic acquired resistance (SAR) responses in plants, we syringe-inoculated two leaves per plant with 10^6^ cfu mL^-1^ of *Pst*/*AvrRpm1* or the corresponding 10 mM MgCl_2_ mock solution. Three days later, two distal leaves were challenged with 10^5^ cfu mL^-1^ of *Pst* by syringe infiltration. Resulting *in planta Pst* titers were evaluated 4 days later as described above.

### Measurement of ROS production

*A. thaliana* ROS production was measured as described (Kutschera et al., 2019). Leaf discs (4 mm) from 8-week-old soil-grown plants were incubated floating on 100 μL water in 96-well white plates in the dark overnight. 30 minutes before the measurements, water in the wells was replaced with 100 μL water with 5 μM L-012 (FUJIFILM Wako chemicals, Japan) and 2 μg/mL horseradish peroxidase (Roche, Switzerland). Luminescence was recorded as relative light units (RLU) in 1 min intervals using a plate reader (Tecan Infinite F200 PRO, Switzerland). After 10 minutes of background measurement, 25 μL of flg22 (final concentration of 100 nM; Pepmic, China) or water as control was added, and measurements were continued for 45 minutes. Data were normalized to average ROS levels 5 min before elicitor application. Average ROS levels of water controls, which were included for each genotype on the same plate, were subtracted for each time point.

### Petiole exudate assays

Petiole exudate (PetEx) were isolated as described (Wenig et al., 2019). Plants were inoculated in two fully expanded leaves with either 10^7^ cfu mL^-1^ of *Pst*/*AvrRpm1*, or with a corresponding 10 mM MgCl_2_ mock solution by syringe infiltration. Simultaneously, additional plants were kept untreated. 24 hours later, the inoculated leaves (or leaves of the same developmental age of untreated plants) were cut in the middle of the rosette. The petioles of six leaves per sample were immersed in 1 mM EDTA for 1h. Subsequently, the EDTA solution was exchanged for 2 mL of sterilized water, and PetEx were collected for 48 h in the dark. Afterwards, PetEx were filter-sterilized (Millipore, 0.22 µm), supplemented with MgCl_2_ to a final concentration of 1 mM, and syringe-infiltrated into fully expanded leaves of naïve recipient plants. 24 hours later, PetEx-treated leaves were either analyzed by qRT-PCR as described below or syringe-infiltrated with 10^5^ cfu mL^-1^ of *Pst.* Resulting *in planta Pst* titers were determined 4 days post infiltration (dpi) as described above.

### Plant-to-plant interaction assays

PTP assays were performed as described (Wenig et al., 2019) in 5.5 L glass vacuum desiccators (Rotilabo-Glas-Exsikkatoren, Roth). 12 Sender plants (in 4 pots) were spray-inoculated with either 10^8^ cfu mL^-1^ *Pst*/*AvrRpm1*, or with 10 mM MgCl_2_ containing 0.01% (v:v) Tween-20 as the corresponding mock control. As an additional control, further sender plants were kept untreated. The sender plants were co-incubated with eight naïve receiver plants (in 2 pots) for 3 days. At 24-hour intervals, the lids of the desiccators were lifted to allow air exchange and release of excess humidity. After co-incubation, fully expanded leaves of the receiver plants were syringe-infiltrated with 10^5^ cfu mL^-1^ of *Pst*, and monitored for *in planta* titers at 4 dpi as described above.

### Xylose-induced resistance assays

To determine systemic defense responses after a chemical induction with xylose, the first fully expanded leaves per plant were syringe-infiltrated with either a specific xylose dose (D-/L-Xylose (ChemCruz™ Biochemicals), D-Xylose, or L-Xylose (Acros Organics)) as indicated, or a corresponding 10 mM MgCl_2_-solution as the mock control. Three days later, two systemic leaves per plant were inoculated with 10^5^ cfu mL^-1^ of *Pst* by syringe infiltration. Resulting *in planta Pst* titers were determined as described above at 4 dpi to assess xylose-induced resistance.

In order to analyze the effect of xylose on bacterial growth rates, we grew 10^7^ cfu mL^-1^ of *Pst* in NYGA liquid medium. This was performed in the wells of 96-well plates, which were supplemented with defined concentrations of D-/L-xylose ranging from 0.1 µM to 1 mM. During a 22 h incubation while shaking (306 rpm) (Tecan INFINITE M1000 PRO), the OD_600_ of the bacterial suspensions was monitored every 20 seconds as a measure of bacterial density/growth.

### RNA isolation and gene expression analysis by qRT-PCR

RNA was isolated with Tri-Reagent (Sigma-Aldrich) according to the manufacturer’s instructions. RNA concentration was determined, and cDNA generated on defined RNA amounts by using SuperScriptII reverse transcriptase (RT; Invitrogen, Thermo Fisher). qPCR was performed on a 7500 Fast real-time qPCR system (Applied Biosystems, Thermo Fisher) with the SensiMix SYBR low ROX kit (Bioline, Meridian Bioscience) and with primers listed in **Supplementary Table S2** and from (Breitenbach et al., 2014; Bauer et al., 2021). Transcript accumulation was analyzed with the Real Quantification 7500 Fast System Software 1.5.1 (Applied Biosystems) and normalized to that of the reference gene *UBIQUITIN*.

### Metabolite profiling by coupled gas chromatography-mass spectrometry

Metabolite analyses were performed essentially as described with minor modifications (Roessner et al., 2001; Lisec et al., 2006; Erban et al., 2007). In brief, 30 mg of the freeze-dried and pulverized plant tissue was extracted in 360 µL of methanol containing 138 µg mL^-1^ of internal standards (ribitol, ^13^C sorbitol) while shaking for 15 min (950 rpm, 70°C). After cooling down the sample to room temperature, 200 μL of chloroform and subsequently 400 μL of distilled water was added, followed by vigorous mixing and centrifugation for 15 min (14,000 rpm, 4°C). An aliquot (50 μL) of the upper, polar phase was transferred into a GC-MS vial (Chromatographie Zubehoer Trott) and dried by evaporation for ~3 h at room temperature (SpeedVac). Subsequently, the pellet was resuspended in 20 μL of methoxyaminhydrochloride (20 mg mL^-1^ in pyridine) and derivatized for 90 min at 37°C. After the addition of 40 µL of BSTFA (N, O-Bis[trimethylsilyl]-trifluoroacetamide) containing 10 μL retention time standard mixture of linear alkanes (n-decane, n-dodecane, n-pentadecane, n-nonadecane, n-docosane, n-octacosane, n-dotriacontane), the mix was incubated at 37°C for additional 45 min. One μL of each sample was analyzed using a GC-TOF-MS system (Pegasus HT, Leco, St Joseph, USA). To this end, the metabolites were separated on a 30 m VF-5ms column with a 10 m EZ-Guard column (Agilent, Santa Clara, USA) using an GC (7890A, Agilent, Santa Clara, USA) and an autosampler system (Combi PAL, CTC Analytics AG, Zwingen, Switzerland). Helium was the carrier gas at a constant flow rate of 1 mL min^-1^. The injection temperature of the split/split-less injector was set to 250°C. Transfer line and ion source were constant at 250°C. The initial oven temperature of 70°C was increased to a final temperature of 320°C by a rate of 9°C min^-1^. The transfer line was set to 250°C as well as the ion source where the metabolites got ionized and fractionated by an ion pulse of 70 eV. Mass spectra were recorded at 20 scans per second with an m/z 35-800 scanning range. Chromatograms and mass spectra were subsequently evaluated and edited using ChromaTOF 4.7 and TagFinder 4.1 software (Luedemann et al., 2008). As reference data base the Golm metabolome database (GMD) was used (Kopka et al., 2005). In total three plant pools, consisting of minimum 12 individual plant rosettes per treatment, were used.

### Statistics

All data were evaluated using the statistic software GraphPad Prism Version 9 for Windows (version 9.0.1 (151)). Outliers were excluded according to the result of the Grubb’s outlier test with α=0.05. Normal distribution was attained after log_2_-transformation of the data for qRT-PCR and bacterial titers. The normal distribution of all data sets was verified with the Shapiro-Wilk test with α = 0.01. Finally, data displaying normal distribution were analyzed by one-way ANOVA analysis with Tukey’s multiple testing correction as indicated in the figure legends.

## Results

### *BXL4* in local defense responses

**Figure 1:**
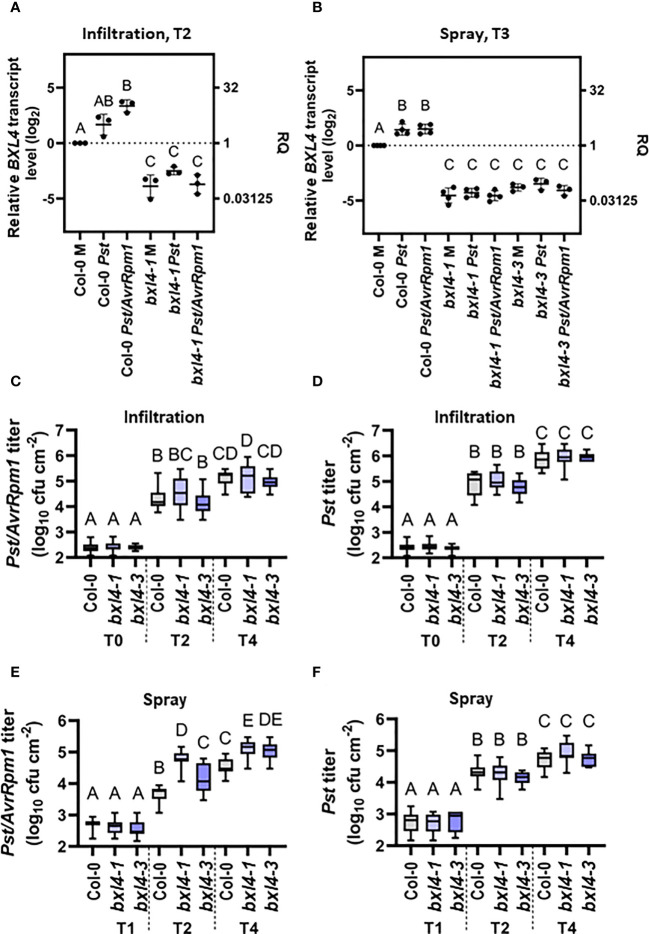
Infection of *Arabidopsis thaliana* with *Pst/AvrRpm1* induces the local accumulation of *BETA*-*D*-*XYLOSIDASE 4* (*BXL4*) transcripts. Col-0, *bxl4-1*, and *bxl4-3* plants were inoculated by syringe infiltration with 10^5^ cfu/mL of *Pst* or *Pst/AvrRpm1* (A/C/D) or by spray treatment with 10^8^ cfu/mL of *Pst* or *Pst/AvrRpm1* (B/E/F). **(A, B)** The *BXL4* transcript abundance in the inoculated leaves was determined by qRT-PCR two (T2) or three days (T3) later. Transcript accumulation was normalized to that of *UBIQUITIN* and is shown relative to the normalized transcript levels in the appropriate Col-0 mock (M) controls. Black dots represent biologically independent data points and horizontal lines represent mean values ± SD from three to four biologically independent replicate experiments. The letters above the scatter dot plots indicate statistically significant differences (one-way ANOVA and Tukey’s test, P=<0.05, for (*BXL4*: infiltration T2): n=3, F(5, 12)=52.31; for (*BXL4*: spray T3): n=3-4, F(8, 24)=125.8). **(C–F)** *In planta* titers of *Pst* and *Pst/AvrRpm1* were determined at the time points indicated below the panels at T0: two hours post inoculation/T1: one day post inoculation (dpi), T2: two dpi, and T4: four dpi. Box plots represent average titers from four to seven biologically independent experiments, including at least three replicates each ± min and max values. Different letters above the box plots indicate statistically significant differences for single means (one-way ANOVA and Tukey’s tests for P=<0.05, for **(C)**: F(8, 136)=144.3, Col-0 T0 n=15, Col-0 T2 n=17, Col-0 T4 n=22, *bxl4-1* T0 n=15, *bxl4-1* T2 n=18, *bxl4-1* T4 n=22, *bxl4-3* T0 n=12, *bxl4-3* T2 n=12, *bxl4-3* T4 n=12; for **(D)**: F(8, 137)=361.6, Col-0 T0 n=15, Col-0 T2 n=18, Col-0 T4 n=22, *bxl4-1* T0 n=15, *bxl4-1* T2 n=18, *bxl4-1* T4 n=22, *bxl4-3* T0 n=12, *bxl4-3* T2 n=12, *bxl4-3* T4 n=12); for **(E)**: F(8, 132)=285.3, Col-0 T1 n=21, Col-0 T2 n=27, Col-0 T4 n=32, *bxl4-1* T1 n=20, *bxl4-1* T2 n=27, *bxl4-1* T4 n=32, *bxl4-3* T1 n=9, *bxl4-3* T2 n=12, *bxl4-3* T4 n=12; for **(F)**: F(8, 164)=169.7, Col-0 T1 n=18, Col-0 T2 n=24, Col-0 T4 n=28, *bxl4-1* T1 n=18, *bxl4-1* T2 n=24, *bxl4-1* T4 n=28, *bxl4-3* T1 n=9, *bxl4-3* T2 n=12, *bxl4-3* T4 n=12).

Breitenbach et al. (2014) reported elevated BXL4 protein levels in the apoplast of *A. thaliana* leaves expressing the *Pseudomonas syringae* (*Ps*) effector *AvrRpm1* when comparing wild type (wt) to *eds1-2* mutant plants. Here, we set out to investigate the role of *BXL4* in innate defense responses of plants. First, we monitored the transcript accumulation of *BXL4* in leaves of Col-0 wt plants after inoculation with virulent *Pst* and with avirulent *Pst/AvrRpm1.* Leaves of 4-5 week old plants were syringe-infiltrated or sprayed with either *Pst*, *Pst/AvrRpm1*, or a corresponding mock solution. *BXL4* transcript abundance was determined at two or three days post inoculation (dpi) depending on the method of inoculation. Two days after infiltration inoculation of Col-0 plants with *Pst*/*AvrRpm1*, *BXL4* transcript levels were elevated as compared to those in mock-treated plants (**Figure 1A**). A treatment with *Pst*, however, caused no significant change in *BXL4* transcript accumulation (**Figure 1A**). The data thus suggest that AvrRpm1 effector-associated responses promote *BXL4* transcript accumulation in wt plants, whereas infection with virulent *Pst* has no significant effect. By contrast, *BXL4* transcript accumulation was moderately induced in leaves of spray-inoculated Col-0 plants in response to both *Pst* and *Pst/AvrRpm1* (**Figure 1B**). These findings suggest that *Pst/AvrRpm1*-infiltrated plants respond with a stronger induction of *BXL4* transcripts than after spray treatment (**Figures 1A, B**). Also, a moderate induction of *BXL4* transcript accumulation after inoculation of the plants with *Pst* cannot be excluded.

In the following, we employed two independent T-DNA insertion alleles of *bxl4*, which we refer to as *bxl4-1* and *bxl4-3*, and inoculated these by spray or infiltration inoculation with either *Pst*, *Pst/AvrRpm1*, or a corresponding mock solution. *BXL4* transcript levels were reduced in both of the *bxl4* lines as compared to Col-0 and did not respond to either of the treatments applied (**Figures 1A, B**).

Subsequently, we investigated whether immune responses towards a bacterial infection with *Pst* or *Pst*/*AvrRpm1* depended on *BXL4*. To this end, we monitored the *in planta* bacterial titers after infiltration inoculation of Col-0 and *bxl4* plants at the day of infection and at 2 and 4 dpi. We detected an increase in bacterial densities over time for both strains in each of the three plant genotypes (**Figures 1C, D**). The rise in titers of both bacterial strains was comparable in wt and *bxl4* mutant plants, suggesting that after syringe-inoculation basal immunity against *Pst* and *Pst/AvrRpm1* was not regulated in a *BXL4-*dependent manner (**Figures 1C, D**). This was further supported by the fact that differences were not detectable in the infection-induced regulation of known defense-related genes, including *PR1* and *LLP1* in *bxl4-1* compared to wt plants (**Supplementary Figures 1A, B**, infiltration, T2). By contrast, spray inoculation of Col-0 and the two *bxl4* mutants with *Pst* or *Pst/AvrRpm1* did reveal differences in bacterial growth when we compared titers at 1, 2, and 4 dpi (**Figures 1E, F**). In contrast to *Pst*, which grew to similar titers in all genotypes (**Figure 1F**), *Pst/AvrRpm1* grew to significantly higher titers at 2 and 4 dpi in both *bxl4* mutants as compared to Col-0 plants (**Figure 1E**). However, transcript accumulation of *PR1* and *LLP1* at 3 days post spray inoculation of the plants treated with *Pst/AvrRpm1* did not appear to be regulated in a *BXL4-*dependent manner (**Supplementary Figures 1A, B**, spray, T3).

Although we did not observe differences in the titers of virulent *Pst* after spray inoculation of wt and *bxl4* mutant plants (**Figure 1F**), this particular interaction appeared to induce *PR1* transcript accumulation to higher levels in *bxl4* mutant as compared to wt plants (**Supplementary Figure 1A**). To test if this might be a consequence of altered PTI, we tested the accumulation of ROS after exposure of leaf discs from wt and *bxl4-1* plants to the PAMP flagellin-22 (flg22). A ROS peak was observed at the same time after flg22 exposure of wt and *bxl4-1* plants and was significantly higher in the *bxl4* mutant than in wt plants (**Supplementary Figure 2**). Together, the data suggest that *BXL4* inhibits local molecular PTI responses, which, however, is not sufficient to significantly alter the propagation of virulent *Pst* bacteria in or on plant leaves. Although we cannot exclude a possible function of *BXL4* in stomatal or cuticular (ETI-associated) defenses against *Pst/AvrRpm1*, ETI against the same pathogen appears to become fully independent of *BXL4* after the pathogen passes the cuticular and/or stomatal barrier (i.e. after syringe infiltration).

### *BXL4* is essential for SAR

**Figure 2:**
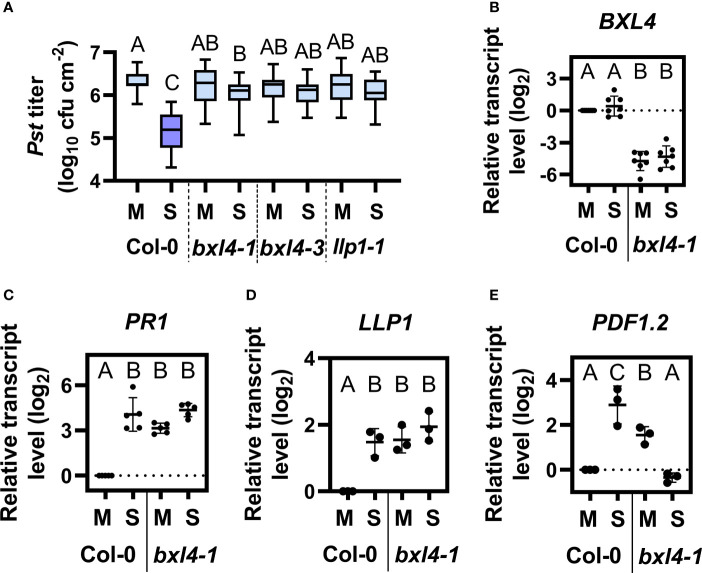
*BXL4* promotes systemic acquired resistance (SAR). Plants of the genotypes Col-0, *bxl4-1*, *bxl4-3*, and *llp1-1* (as indicated below the panels) were syringe-infiltrated in the first two true leaves with either 10^6^ for **(A–C)** or 10^7^ (for D/E) cfu per mL of *Pst/AvrRpm*1 (S) or a corresponding mock (M) control solution. Distal uninfected leaves were examined for the transcript abundance of *BXL4* (**B**, at 3 dpi), *PR1* (**C**, at 3 dpi), *LLP1* (**D**, at 1 dpi), or *PDF1.2* (**E**, at 1 dpi). Alternatively, plants were challenged at 3 dpi with 10^5^ cfu/mL of *Pst* to evaluate SAR. **(A)** *In planta* titers of *Pst* in systemic, challenge-inoculated leaves were measured at 4 dpi. Box plots represent average *Pst* titers from nine biologically independent experiments, including at least 3 replicates each ± min and max values. Letters above the box plots indicate statistically significant differences for means (one-way ANOVA and Tukey’s test for P=<0.05, F (28, 403)=21.02, Col-0 M n=33, Col-0 S n=37, *bxl4-1* M n=40, *bxl4-1* S n=42, *bxl4-3* M n=21, *bxl4-3* S n=22, *llp1-1* M n=25, *llp1-1* S n=25). **(B–E)** Transcript abundance of the genes was measured by qRT-PCR, normalized to that of *UBIQUITIN*, and is shown relative to the normalized transcript levels in the appropriate Col-0 mock (M) controls. Black dots represent three to seven biologically independent data points, and lines indicate the respective mean values ± SD. The letters above the scatter dot plots indicate statistically significant differences (one-way ANOVA and Tukey’s test, P=<0.05, for **(B)**: n=7, F(5, 32)=68.60, for **(C)**: n=5, F(5, 24)=20.68, for **(D)**: n=3, F(3, 8)=29.80, for **(E)**: n=3, F (3, 8)=16.74).

Because *BXL4* had previously been identified in a screen for SAR-associated proteins (Breitenbach et al., 2014), we next investigated if *BXL4* modulates responses to a secondary bacterial challenge in distal tissues. To this end, we monitored systemic immunity to *Pst* in wt and *bxl4* mutant plants after a local stimulus. First, we inoculated the first two true leaves of Col-0, *bxl4-1*, *bxl4-3* with either a SAR stimulus (here: *Pst*/*AvrRpm1*) or a corresponding mock solution. Because the primary treatment was performed by syringe infiltration, local responses to the SAR trigger *Pst/AvrRpm1* were comparable in wt and *bxl4* mutant plants (**Figure 1C** and **Supplementary Figure 1**). We further included *llp1-1* mutant plants with a known SAR-defective phenotype as control (Breitenbach et al., 2014). Three days after the primary stimulus, distal leaves were challenged with virulent *Pst*, again by syringe infiltration, and monitored for *in planta* bacterial titers 4 days later. The results showed that SAR-induced Col-0 displayed reduced *Pst* titers as compared to mock-treated plants (**Figure 2A**), indicating an activated resistance (SAR) against the bacterial challenge. In contrast, both *bxl4* mutant genotypes supported similar bacterial densities in both *Pst/AvrRpm1*-stimulated and control-treated plants (**Figure 2A**), indicating that SAR could not be activated in these plants. In support, *Pst-*induced symptoms were more severe on the systemic leaves of SAR-activated *bxl4-1* mutant as compared to wt plants (**Supplementary Figure 3**). Consequently, as previously shown for *LLP1*, the data strongly suggest that *BXL4* is essential for SAR-associated systemic immunity in response to a local *Pst/AvrRpm1* inoculation.

We then went on to examine the transcript abundance of stress-associated genes in systemic leaves of Col-0 and *bxl4-1* plants 3 days after a local SAR stimulus or a mock treatment. *BXL4* transcript accumulation was not changed in the systemic tissues of SAR-induced Col-0 (**Figure 2B**). However, the transcript level of *PR1* was promoted in Col-0 after SAR induction and further leveled the same as in *bxl4-1* after any treatment (**Figure 2C**). We additionally determined the transcript accumulation of *LLP1* and *PDF1.2* in systemic tissues 1 day after a local SAR stimulus or mock treatment. The expression of *LLP1* was induced in Col-0 after the bacterial infection, and moreover upregulated in *bxl4-1* after both treatments (**Figure 2D**). These findings suggest that *PR1* and *LLP1* transcript accumulation might be similarly regulated in a *Pst/AvrRpm1-*dependent and *BXL4-*modulated manner. As shown previously, the establishment of SAR involves an early systemic induction of the JA pathway (Truman et al., 2007). Here, transcript accumulation of the JA marker gene *PDF1.2* was induced systemically during SAR in Col-0 plants one day after the SAR stimulus, whereas such a response was absent in *bxl4-1* (**Figure 2E**). This suggests that an early systemic induction of JA responses during SAR depends on *BXL4.*

Notably, the transcript accumulation of both SA (*PR1*) and JA marker genes (*PDF1.2*) as well as that of SAR-associated *LLP1* was elevated in the systemic tissues of mock-treated *bxl4-1* as compared to wt (**Figures 2C–E**). Although this phenomenon was not associated with heightened systemic resistance against *Pst* (**Figure 2A**), we cannot exclude a minor influence of *BXL4* on defense in response to a local mock treatment. Because the accumulation of the respective defense-associated transcripts was not further induced in *bxl4-1* plants during SAR (and in the case of *PDF1.2* was markedly reduced, **Figure 2E**), the data support the hypothesis that *BXL4* is important for SAR-associated molecular responses in *A. thaliana.*

### Long-distance SAR signaling *via* the phloem depends on BXL4

Stress signals produced at the site of inoculation can be transmitted towards distal parts within a plant *via* the phloem or can be emitted and move from leaf to leaf (or from plant to plant) *via* the air. As *BXL4* is transcriptionally regulated after *Pst/AvrRpm1* in local, infected tissues (**Figure 1A**) and is necessary for SAR (**Figure 2A**), we tackled the question if *BXL4* contributes to long-distance signaling in SAR.

**Figure 3:**
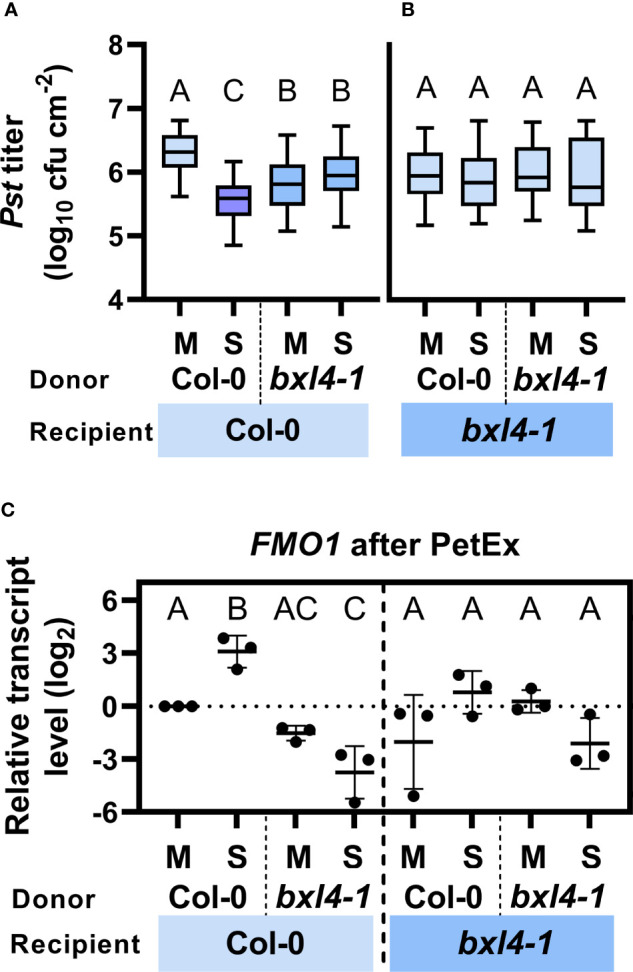
Both local generation/transmission and systemic perception/propagation of phloem-mobile SAR signals depends on *BXL4*. Leaves of Col-0 and *bxl4-1* plants were inoculated with either 10^7^ cfu/mL of *Pst/AvrRpm1* (S) or a corresponding mock (M) solution. One day later, petiole exudates (PetEx) were collected from the inoculated leaves and syringe-infiltrated into leaves of naïve Col-0 and *bxl4-1* recipient plants. One day later, the inoculated recipient leaves were either collected for qRT-PCR analysis or challenged with 10^5^ cfu/mL of *Pst*. **(A, B)** Bacterial titers in challenge-inoculated leaves of receiver plants were monitored at 4 dpi. Box plots represent average *Pst* titers in Col-0 **(A)** and *bxl4-1* **(B)** recipient plants from four to twelve biologically independent experiments ± min and max values. Letters above the box plots indicate statistically significant differences for means (one-way ANOVA and Tukey’s test for P=<0.05, for (**A**, Col-0 recipients): F(3, 191)=35.07, Col-0 M n=51, Col-0 S n=48, *bxl4-1* M n=49, *bxl4-1* S n=47; for (**B**, *bxl4-1* recipients): F(3, 116)=0.4254, Col-0 M n=45, Col-0 S n=44, *bxl4-1* M n=16, *bxl4-1* S n=15). **(C)** Transcript abundance of *FLAVIN-DEPENDENT MONOOXYGENASE 1* (*FMO1*) was determined by qRT-PCR, normalized to that of *UBIQUITIN*, and is shown relative to the normalized transcript levels of the appropriate Col-0 mock (M) control. Black dots represent three biologically independent data points and lines indicate the respective mean values ± SD. The letters above the scatter dot plots indicate statistically significant differences (one-way ANOVA and Tukey’s test for P=<0.05, n=3, F (5, 12)=17.77).

We initially investigated if *BXL4* modulates the generation of SAR signals and the transmission of molecules *via* the phloem-mobile route. To this end, we performed petiole exudate (PetEx) experiments with Col-0 and *bxl4-1* plants. We stimulated plants for the generation of phloem sap-associated molecules by syringe-inoculation of either *Pst/AvrRpm1* or a corresponding mock solution. One day later, we collected PetEx from the inoculated (donor) leaves and also from (donor) plants that had been kept untreated. To evaluate the SAR-inducing capacities of PetEx, we infiltrated these into naïve Col-0 and *bxl4-1* recipient plants, and one day later challenged the same leaves with *Pst*. *In planta Pst* titers were monitored at 4 dpi. In these experiments the PetEx of bacteria-infected Col-0 donors reduced the propagation of the *Pst* challenge inoculum in Col-0 recipients, thus rendering these plants more resistant against *Pst* compared to recipient plants, which had been treated with PetEx of control-treated wt plants (**Figure 3A**). Notably, PetEx from both mock- and *Pst/AvrRpm1*-treated *bxl4-1* donors reduced the *Pst* titers in Col-0 recipient plants but were neither as effective as PetEx from infected wt plants (**Figure 3A**). These findings suggest that *BXL4* is necessary for SAR signal generation or transmission from local, infected tissues. Also, it is possible that the mock treatment of donor leaves caused a minor induction of defense-associated signals in the PetEx of *bxl4-1* plants, which – during normal systemic signaling – might in turn be causative for a systemic elevation of defense gene expression (**Figures 2C–E**). Nevertheless, further SAR signals in response to *Pst/AvrRpm1* appeared to be lacking from *bxl4-1* PetEx (**Figure 3A**).

We next asked ourselves if *bxl4-1* recipient plants could respond to SAR signals in PetEx. To this end, we analyzed *Pst* growth in *bxl4-1* recipient plants, which had been inoculated with *Pst* one day after their treatment with the same PetEx as above. No differences in *Pst* growth were detected in *bxl4-1* recipients in response to any of the PetEx (**Figure 3B**), suggesting that *BXL4* is essential for the recognition or propagation of phloem-mobile defense signals in the systemic tissues during SAR.

Because PetEx from mock-treated *bxl4-1* donors appeared to induce a moderate defense response in wt, but not *bxl4-1* recipient plants (**Figures 3A, B**), we also investigated if *bxl4-1* donor plants perhaps constitutively accumulated defense-modulating compounds in exudates of leafy tissues. To address this question, we used PetEx from untreated wt and *bxl4-1* donor plants and treated recipients of both genotypes. The recipients were inoculated with *Pst* one day after the PetEx treatment. No differences could be observed in the *Pst* densities at 4 dpi of wt or *bxl4-1* recipient plants in response to PetEx from untreated wt or *bxl4-1* donors (**Supplementary Figure 4**). This data suggests that *bxl4-1* donors accumulate mock inoculation/wounding-inducible molecules in defense-promoting PetEx (**Figure 3A**) rather than constantly expressing SAR-promoting compounds. This supports the above observation of enhanced *PR1* expression in systemic, but not local *bxl4-1* tissues after *Pst/AvrRpm1* infection (**Figure 2C** and **Supplementary Figure 1A**).

Together, the data suggest that BXL4 might be part of a homeostatic feedback system to avoid the release of defense-inducing signals in the absence of infection. More importantly, the data confirm that BXL4 likely promotes SAR both locally, during SAR signal generation or transmission, and systemically in SAR signal recognition or propagation in response to a local *Pst/AvrRpm1* infection.

### *BXL4* putatively regulates *FMO1* during SAR

Because BXL4 is an important modulator of SAR signals, we aimed to identify possible pathways that are associated with *BXL4* and SAR. Similarly to *BXL4*, Pip/NHP has been associated with both local SAR signal generation/transmission (Wang et al., 2018b; Jiang et al., 2021) and systemic SAR signal recognition/propagation (Wang et al., 2018a). *FMO1* is induced systemically during SAR and codes for the enzyme which converts Pip into its bio-active derivative NHP (Mishina and Zeier, 2006; Chen et al., 2018; Hartmann et al., 2018). Here, we analyzed the transcript abundance of *FMO1* in PetEx-treated leaves of Col-0 and *bxl4-1* recipient plants at 1 dpi. We found that *FMO1* transcript accumulation was induced upon treatment of wt recipient plants with PetEx from infected wt, but not *bxl4-1* plants (**Figure 3C**). In fact, *FMO1* transcript accumulation was reduced in wt recipients of *bxl4-1-*derived PetEx and this became significant after treatment of the plants with PetEx from SAR-activated *bxl4-1* donor plants. This data suggests that *BXL4* modifies phloem-mobile signals that promote a *Pst/AvrRpm1*-related transcript accumulation of *FMO1* in PetEx-treated wt recipients. Reciprocally, *bxl4-1* plants also did not respond with enhanced *FMO1* transcript accumulation to the PetEx of SAR-activated wt plants (**Figure 3C**). In sum, *BXL4* might contribute to fortify *FMO1*- and thus Pip/NHP-associated responses during SAR signaling.

### *BXL4* promotes communication *via* the airborne route

**Figure 4:**
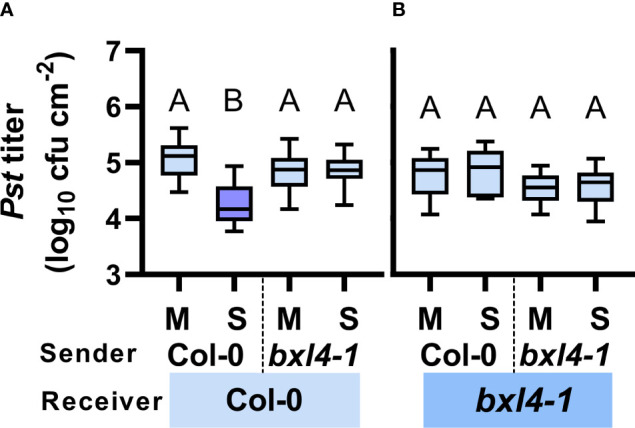
Both local generation/emission and systemic perception/propagation of airborne SAR signals depends on *BXL4*. Col-0 and *bxl4-1* sender plants were spray-inoculated with either 10^8^ cfu/mL of *Pst/AvrRpm1* (S) or a corresponding mock (M) solution. Sender plants were co-incubated in desiccators with naïve receiver plants. Three days later, leaves of receiver plants were inoculated with 10^5^ cfu/mL of *Pst*. The resulting *in planta Pst* titers in Col-0 **(A)** and *bxl4-1* **(B)** receiver plants were evaluated at 4 dpi. Box plots represent average *Pst* titers from four to seven biologically independent experiments, including at least 3 replicates each ± min and max values. Letters above the box plots indicate statistically significant differences for means (one-way ANOVA and Tukey’s test for P=<0.05, for (A, Col-0 receivers): F(3, 118)=37.26, Col-0 M n=30, Col-0 S n=32, *bxl4-1* M n=30, *bxl4-1* S n=30; for (B, *bxl4-1* receivers): F(3, 63)=3.383, Col-0 M n=17, Col-0 S n=17, *bxl4-1* M n=16, *bxl4-1* S n=17).

Due to the putatively multifaceted interactions of *BXL4* in signaling *via* the phloem-mobile route, we speculated that *BXL4* might also interfere with inter-plant interaction *via* volatiles. We therefore tested, if signaling by airborne signals between ETI-infected sender plants and healthy receivers was dependent on *BXL4*. To address this, we spray-inoculated Col-0 and *bxl4-1* sender plants with either *Pst*/*AvrRpm1* or a corresponding mock solution. Subsequently, senders were co-incubated with naïve receiver plants in closed desiccators to enforce a directed communication. The desiccators were opened once every 24 hours to let in fresh air. After three days, the receiver plants were challenged with *Pst* and monitored for bacterial densities 4 days later. When receivers exhibited lower titers of *Pst* than the corresponding controls, we considered the receivers to recognize and respond to airborne molecules from emitting senders. In turn, when receivers did not react with bacterial densities distinct from the control plants, the senders were either not producing or transmitting defense-inducing volatiles. Here, Col-0 receivers, which shared air space with infected wt senders, responded with reduced *Pst* titers as compared to Col-0 plants co-incubated with mock-treated wt senders (**Figure 4A**). Nonetheless, when wt receivers were exposed to *bxl4-1* senders, no differences were detected in titers of *Pst*. Therefore, the data suggest that *BXL4* is necessary for the generation of SAR-related volatile signals and by this for defense against *Pst*. Subsequently, we tested *bxl4-1* receiver plants and detected no differences in *Pst* titers after plants were co-incubated with any of the sender plants (**Figure 4B**). Consequently, we propose that *BXL4* is necessary for the perception of airborne molecules in receiver plants. Taken together, *BXL4* might activate multiple layers of ETI defense that are relevant for SAR, promoting both SAR signal generation and perception in intra- and inter-plant interactions (**Figures 3A**, **4A**). This further leads to the question, which actions of BXL4 proteins *in planta* might affect the signaling routes involved.

### Exogenous xylose triggers SAR-associated immune responses in *A. thaliana*

**Figure 5:**
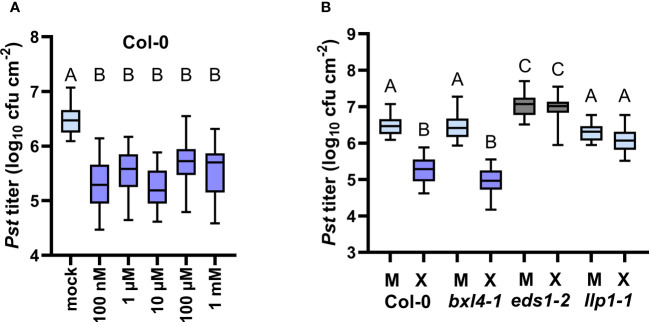
Exogenous xylose induces *EDS1*- and *LLP1*-dependent systemic defense against *Pst*. Plants of the genotypes Col-0, *bxl4-1*, *eds1-2*, and *llp1-1* were inoculated with a mixture of D- and L-xylose, termed D-/L-xylose (10 µM (X) or another dose as indicated below the panels), or with a corresponding mock treatment (M or mock as indicated below the panels). Three days later, two distal leaves were challenged with 10^5^ cfu/mL of *Pst* and monitored for *in planta* bacterial titers 4 days after. Box plots represent average *Pst* titers of 12 **(A)** or seven **(B)** biologically independent experiments ± min and max value. Different letters above box plots indicate statistically significant differences for means (one-way ANOVA and Tukey’s test for P=<0.05, for (**A**, Col-0): F(5, 189)=46.95, mock n=46, 100 nM n=34, 1 µM n=27, 10 µM n=35, 100 µM n=27, 1 mM n=26; for **(B)**: F(7, 284)=43.20, Col-0 M n=46, Col-0 X n=35, *bxl4-1* M n=46, *bxl4-1* X n=30, *eds1-2* M n=41, *eds1-2* X n=25, *llp1-1* M n=43, *llp1-1* X n=26).

As introduced above, BXL4 was previously shown to act as a functional β-xylosidase *in planta* where BXL4 putatively hydrolyses polysaccharides such as xylan whose backbone mainly consist of xylose molecules (Minic et al., 2004). We thus hypothesized that BXL4 potentially promotes the release of xylose-associated molecules from cell walls, thereby modulating xylose levels (or those of xylose derivatives) in tissues of (ETI) defense-induced plants. Therefore, we assessed how exogenously applied xylose affects plant immune responses. In order to test this, we inoculated Col-0 and SAR-defective mutant plants, including *bxl4-1, eds1-2*, and *llp1-1* (Breitenbach et al., 2014), with either D-/L-xylose or a corresponding mock solution. At 3 dpi we challenged systemic leaves with *Pst* and examined the bacterial titers 4 days later. Here, wt plants which were pretreated with a dose of 100 nM up to 1 mM of D-/L-xylose mounted reduced *Pst* titers when compared to the control-treated plants (**Figure 5A**). This indicates an effective, dose-independent induction of plant resistance by xylose, which was comparable to responses induced by either D- or L-xylose alone (**Supplementary Figure 5**). Interestingly, *bxl4-1* responded with comparable *Pst* titers as Col-0 plants after a treatment with 10 µM of D-/L-xylose (**Figure 5B**), suggesting that BXL4 acts either upstream of xylose in xylose-induced resistance or in a parallel, independent pathway.

In a natural environment, diverse bacteria can perceive and take up D-xylose (Li et al., 2017) or degrade xylose enzymatically by xylose isomerases (Feil et al., 2005). We thus investigated if growth of *Pst*, as used in our assays, could be affected by exogenous xylose. To this end, we cultivated *Pst* in liquid culture supplemented with either a xylose dose or a corresponding mock solution, and monitored bacterial growth over the course of 22 h. *Pst* bacteria grew to similar densities in the presence of xylose as compared to the control treatment (**Supplementary Figure 6**), indicating that xylose does not restrict or promote *Pst* growth *in vitro*. We thus propose that the growth of *Pst* bacteria *in planta* is not affected by exogenous xylose, which puts forward the hypothesis that xylose-induced defense responses, as described above (**Figure 5A**), depend on the interaction of the plant with *Pst*. In support of this, while *Pst* titers in *eds1-2* mutant plants were elevated as a result of compromised SA defenses in these plants (Falk et al., 1999), the xylose treatment did not cause a reduction of *Pst* growth in *eds1-2* (**Figure 5B** and **Supplementary Figure 5**). Similarly, *Pst* titers in *llp1-1* were the same in xylose- and mock-treated plants, suggesting that xylose-induced resistance depends on the plant defense components *EDS1* and *LLP1.* At the same time, we observed a moderate, but insignificant induction of *PR-1* transcript accumulation in xylose-treated wt plants, while *PR-1* transcripts remained unchanged in *bxl4-1* mutants (**Supplementary Figure 7**). Together, these data suggest that exogenous xylose induces resistance in *A. thaliana* by acting through a defense pathway that is associated with *EDS1* and *LLP1*.

### *BXL4* acts upstream of a variety of defense-associated small molecules

Because *bxl4* mutant plants should contain less soluble xylose and potentially more xylose that is bound in cell wall glycan structures, it appears that the *bxl4* phenotypes reported above are not an immediate result of the release of xylose residues from cell walls into the apoplast. Notably, *AvrRpm1*-inducible responses are suggested to trigger pathways involved in the modulation of carbohydrate metabolism (Gao et al., 2020). We thus asked if polar, soluble metabolites and carbohydrate-related molecules aside from cell wall glycans are regulated in an *AvrRpm1*-related and *BXL4*-dependent manner in *A. thaliana*. To this end, we spray-inoculated Col-0, *bxl4-1*, and as a control also *eds1-2* plants with either *Pst*/*AvrRpm1* or a corresponding mock solution. Two days later, the rosettes of 12 plants per genotype were pooled per sample and rapidly frozen in liquid nitrogen to analyze profiles of polar and semi-polar metabolites by GC-MS. In total, we detected 137 different metabolites, some of which accumulated differentially in both *bxl4-1* and *eds1-2* plants as compared to wt (**Supplementary Table 3**). These included fucose, G3P, putrescine, and serine (**Table 1**). Of these, fucose, G3P, and serine displayed elevated relative abundances in mock-treated *bxl4-1* plants as compared to wt with moderate to no further change after *Pst/AvrRpm1* inoculation. The same compounds displayed significantly induced relative abundances in infected *eds1-2* plants as compared to mock-treated wt (**Table 1** and **Supplementary Table 3**). The relative abundance of putrescine was elevated after *Pst/AvrRpm1* inoculation of both *bxl4-1* and *eds1-2* but not wt plants. Further, the relative abundance of raffinose was elevated in *eds1-2* mutant plants irrespective of the treatment, while that of glucose-6-phosphate was elevated in *bxl4-1* mutants (**Supplementary Table 3**). Also, both 4-hydroxy-butanoic acid and diethylenglycol were upregulated in *bxl4-1* plants after infection when compared to wt. In the *eds1-2* mutant, glucose and ornithine accumulated to higher than wt levels in infected plants, while the accumulation of threonine was reduced in mock-treated *eds1-2* as compared to wt plants. Together, these results indicate that *BXL4* and *EDS1* affect the levels of specific sugars, amino acids and polyamines *via* individual and/or mutually stimulated plant responses upon an ETI/SAR trigger.

**Table 1:** Relative abundance of metabolites, which displayed significant differences to wt in both *bxl4-1* and *eds1-2* mutant plants.

Metabolite	Col-0 M	Col-0 R	*bxl4-1* M	*bxl4-1* R	*eds1-2* M	*eds1-2* R
Fucose	1.000	1.30 ± 0.29	**2.19 ± 1.89**	**2,22 ± 1.49**	1.18 ± 0.80	**2.42 ± 1.50**
G3P	1.000	1.43 ± 0.19	**2.07 ± 0.92**	**2.36 ± 1.24**	1.26 ± 0.82	**2.05 ± 1.37**
Putrescine	1.000	1.08 ± 0.13	1.09 ± 0.35	**2.03 ± 0.92**	0.78 ± 0.02	**2.54 ± 1.77**
Serine	1.000	1.43 ± 0.38	**2.00 ± 0.29**	**2.43 ± 0.42**	**1.90 ± 1.18**	**3.60 ± 1.66**

Plants were inoculated with *Pst/AvrRpm1* or a corresponding mock control treatment and metabolites were analyzed by GC-MS in the soluble fractions of cell wall extracts taken at 2 dpi. Data represent the average abundance normalized to sample dry weight and relative to mock-treated Col-0 wt plants from three biologically independent replicates ± SD. **Values in bold** are significantly different from mock-treated Col-0 wt (two-way ANOVA with Bonferroni’s multiple comparison test or False Discovery Rate (FDR) with two-stage linear step-up procedure of Benjamini, Krieger and Yekutieli; P=<0.05, *p*-values are listed in **Supplementary Table 3**).

## Discussion

This work identifies BXL4 and thus the cell wall and BXL4-associated cell wall dynamics as an important, new regulatory component of SAR. The fact that BXL4 acts upstream of both SAR signal generation in local, infected tissues and of SAR signal recognition/propagation in systemic leaves strongly suggests that BXL4 acts early in SAR signaling cascades (**Figure 3**). This is further corroborated by the fact that BXL4 action in SAR influences both phloem-mediated and airborne systemic signaling (**Figures 3**, **4**), while BXL4 also acts upstream of or in parallel with xylose in induced resistance (**Figure 5**). Consequently, the cell wall might play a central role in the regulation and/or establishment of SAR. Stomata, cell walls and the apoplastic space are probable areas of a plant where plant-pathogenic signals can be initially perceived (Melotto et al., 2017; Saijo et al., 2018; Molina et al., 2021). Upon interaction of a plant with pathogens, downstream responses are launched that can promote transcriptional events and immunity. Interestingly, in spray-inoculated plants, a *BXL4*-associated ETI component was clearly recognizable, whereas such a response was absent after inoculation by syringe infiltration (**Figure 1**). We consequently hypothesized that the method of plant inoculation might modify *BXL4*-associated pathways, including defense. Possibly, such plant responses are related to stomatal defense (Melotto et al., 2017) or wounding-associated signaling (Farmer et al., 2014; Wasternack and Song, 2017). Because a *BXL4-*associated defense component was not observed after spray inoculation of virulent *Pst* (**Figure 1**) and thus was not consistently observed with multiple pathogens, the data exclude a role of BXL4 in stomatal immunity. Rather, wounding as incurred, for example, during syringe infiltration of pathogens, might interfere with *BXL4-*associated ETI responses in syringe-infiltrated as compared to spray-inoculated plants. Plant responses to wounding rely on jasmonate signaling (Farmer et al., 2014; Wasternack and Song, 2017), which is believed to antagonize SA-associated immune responses (Pieterse et al., 2012). Thus, such responses might complement the loss of a BXL4 defense component and obscure local, BXL4-associated ETI phenotypes. In support of this hypothesis, we observed elevated transcript accumulation of e.g. the SAR marker gene *PR-1* in *bxl4-1* plants after syringe infiltration of a mock, control treatment in multiple experiments (**Figure 2**). Also, PetEx of mock-treated, *bxl4-1* donor plants moderately reduced the propagation of a *Pst* challenge inoculum in wt recipient plants (**Figure 3**). Because the same was not observed using PetEx from untreated *bxl4-1* donor plants (**Supplementary Figure 4**), the dissemination of defense-active signals in *bxl4* mutant plants might result from responses to wounding, including those occurring during a mock, control treatment.

Former studies demonstrated that *BXL4* transcripts considerably increased at 2-12 hours after a wounding stimulus (up to 16-fold when compared to the non-stressed state) (Kilian et al., 2007; Guzha et al., 2022). Wounding responses moreover promote the expression of the JA pathway genes *PDF1.2* and *JASMONATE-ZIM-DOMAIN PROTEIN 10* (*JAZ10*) and this response is dependent on *BXL4* (Guzha et al., 2022). Also, *BXL4* partially regulates the synthesis of JA derivatives (such as jasmonoyl-L-isoleucine, JA-Ile) either after an infection with *Botrytis cinerea* or at ~2 hours after wounding (Guzha et al., 2022). Here, an early induction of the JA marker gene *PDF1.2* was detected in the systemic tissues of wt plants during SAR and this depended on *BXL4* (**Figure 2**). Thus, it is possible that BXL4 promotes JA-associated responses to wounding, also early after infection of *A. thaliana* with a hemi-biotrophic bacterium, such as *Pst/AvrRpm1* used here to induce SAR. This might compromise SA defense and ETI due to local antagonistic cross talk between the JA and SA signaling sectors in syringe-infiltrated plants (**Figure 1**). In turn, this might obscure an additional influence of BXL4 on ETI detected in the absence of wounding in plants inoculated with *Pst/AvrRpm1* by spray inoculation (**Figure 1**). Because the SAR-deficient phenotype of *bxl4* mutant plants appeared more robust than local defense phenotypes in response to *Pst/AvrRpm1*, we posit that such an additional function of BXL4 is associated with the establishment of SAR.

The plant cuticle and epidermis have been shown to play essential roles in SAR (Xia et al., 2009; Jiang et al., 2021). Here, we observed a potentially JA- and/or wounding-associated and BXL4-dependent influence on local defense phenotypes after spray inoculation of plants (**Figure 1** and **Supplementary Figure 1**). Also, the flg22-induced ROS burst was slightly enhanced in leaf discs of plants lacking functional BXL4 (**Supplementary Figure 2**). Perhaps BXL4-associated cell wall dynamics at the leaf surface interacts with JA-associated signals to compromise local defense responses and promote systemic immunity.

An effective SAR response depends on the biosynthesis of SA and the accumulation of NHP, both of which processes can be transcriptionally promoted by *EDS1* upon stress (Hartmann and Zeier, 2019). This indicates a key functional role of *EDS1* and associated pathways in the metabolic regulation of SA and NHP derivatives, SAR signaling, and defense. The balance of SA and NHP and their glycosylated derivatives SAG and NHP-H2 is suggested to balance growth and defense establishment in stressed plants (Bauer et al., 2021; Cai et al., 2021; Mohnike et al., 2021). Here, transcript accumulation of *FMO1*, the enzyme which converts Pip into its SAR bioactive derivative NHP (Chen et al., 2018; Hartmann et al., 2018), was induced in recipient plants in response to SAR-activated PetEx (**Figure 3**). Because this was dependent on functional *BXL4* [during SAR signal generation] in the donor plant, these data provide further support to the hypothesis that BXL4 has a central role in SAR signaling.

As BXL4 is a protein that is presumably secreted to the apoplast (Goujon et al., 2003; Breitenbach et al., 2014; Sham et al., 2014; Guzha et al., 2022), its localization close to cell walls and plasma membranes likely determines where it may function (as a putative xylosidase) in plants. Guzha et al. (2022) recently described BXL4 functions in comparison with those of seed coat-associated BXL1 proteins, which are bifunctional β-D-xylosidases/α-arabinofuranosidases (Goujon et al., 2003; Minic et al., 2004) sharing 57% identity with BXL4 at the amino acid level (Arsovski et al., 2009). The data suggest that *BXL4* and *BXL1* both modify the composition of seed mucilage, potentially altering monosaccharide levels in dependence of *BXL1* (arabinose) and *BXL4* (xylose) (Guzha et al., 2022). The authors suggest that BXL4 suppresses the accumulation of xylose in mucilage by modifying RG-I components including xylan and arabinan (Guzha et al., 2022). However, transgenic lines overexpressing *BXL4* accumulated equal amounts of xylose and arabinose in rosette leaves when compared to wt plants (Guzha et al., 2022), suggesting that *BXL4*-inducible plant responses may be regulated in an organ-specific manner and/or in relation to plant development. In support of this, the leaves of *bxl4* mutant plants in our study also did not accumulate reduced free xylose levels when compared to wt plants after infection (**Supplementary Table 3**). In contrast, Guzha et al. (2022) detected elevated levels of, for example, arabinose and fucose in leaf pectin of non-stressed *bxl4* mutants in comparison to wt plants, suggesting that BXL4 may specifically alter pectin structures and the content of cell wall-associated saccharides in leafy tissues.

In our study, free fucose levels were elevated in rosette leaves of *bxl4-1* and *eds1-2* mutant plants (**Table 1** and **Supplementary Table 3**). Because Guzha et al. (2022) essentially detected the same when analyzing leaf pectin, our collective data suggest that *BXL4* signals control fucose retention in leaf pectin structures. As a result, such interlinking of pectin with other components of the cell wall or the breakdown thereof might greatly affect the flexibility and stiffness of cell walls (Bidhendi and Geitmann, 2016). This, in turn, has been proposed to influence the accessibility of these physical barriers for invading pathogens (Bacete and Hamann, 2020). Notably, mutant plants defective in the gene *MURUS1* (also termed *GDP-D-MANNOSE-4,6-DEHYDRATASE 2*) have decreased levels of fucose and fucosylated arabinogalactan proteins (AGP) as well as reduced cross-linkages in pectin (Tryfona et al., 2012; Feng et al., 2018). These *MURUS1*-related alterations in pectin cross-links have been suggested to contribute to PTI, ETI, and additionally stomatal and apoplastic defense (Zhang et al., 2019). Together, the data suggest that modifications of cell wall pectins and fucose levels downstream of BXL4 might also play a role in SAR.

Interestingly, levels of the putative long-distance SAR signal G3P (Chanda et al., 2011) appeared to be regulated in a similar manner as fucose in *eds1* and *bxl4* mutant plants (**Table 1** and **Supplementary Table 3**). Hence, we hypothesize that pathways downstream of *EDS1* and *BXL4* might modulate the abundance of both G3P and fucose (originating from pectin components in cell walls or AGPs) during defense. G3P is one of several putative long-distance signals of SAR (Chanda et al., 2011; Gao et al., 2015; Vlot et al., 2021). Notably, Moradi et al. (2021) described that a local treatment with an exogenously applied fungal, fucose-binding lectin protein induced SAR against *Pst*. Concomitantly, transcripts of genes, including the G3P biosynthetic enzyme *GLY1* as well as *PR-1* and *RESPIRATORY BURST OXIDASE HOMOLOGS D* and *F* (*RBOHD* and *F*) were induced in the lectin-inoculated leaves (Moradi et al., 2021). This suggests that fucose-associated signals may promote the enrichment of G3P while also boosting local ROS signals. Defense signaling *via* RBOHD/F may further be associated with the polyamine putrescine, which was described to promote PTI through apoplastic ROS (hydrogen peroxide) and RBOHD/F (Liu et al., 2019). As we found that putrescine was exclusively upregulated in *Pst*/*AvrRpm1-*inoculated *eds1* and *bxl4* mutant as compared to wt plants (**Table 1** and **Supplementary Table 3**), we hypothesize that EDS1 and BXL4 may have a repressive effect on the accumulation of putrescine during SAR signal generation – potentially also as a result of reduced fucose levels. Notably, high putrescine levels in plants have been associated with defense responses against a variety of stresses, including disease, in plants (Gonzalez-Hernandez et al., 2022). In this respect, it is of interest to note that local resistance responses to *Pst/AvrRpm1* are largely independent of EDS1, while SAR signal generation in the same interaction fully depends on EDS1 (Aarts et al., 1998; Breitenbach et al., 2014). Similarly, local effects of BXL4 on defense against *Pst/AvrRpm1* are minor (**Figure 1** annd **Supplementary Figure 1**), while SAR signal generation and perception fully depend on BXL4 (**Figures 2**–**4**). Thus, our data suggest that EDS1 and BXL4 act in a homeostatic feedback system to limit local defense signaling in favor of systemic dissemination of immune signals. Negative feedback on local immunity in favor of systemic defense might exploit cell wall dynamics associated with fucose and putrescine (**Table 1**).

This is the first report of a central function of cell wall-associated metabolic changes on systemic immunity, in particular SAR. With roles in both local SAR signal generation and in systemic SAR signal perception or propagation, the data suggest that the cell wall plays a central role in mitigating SAR. Further research should contribute to unraveling the molecular interaction between cell wall dynamics and SAR in order to pave the road for the development of novel, cell wall-derived agents for application in sustainable (crop) plant protection measures.

## Data availability statement

The original contributions presented in the study are included in the article/**Supplementary Material**. Further inquiries can be directed to the corresponding author.

## Author contributions

KB and ACV conceived the project. KB, ML, MW, SR, PG, and ACV planned experiments. KB, SN, ML, MW, and L-JS performed experiments and analyzed the data. KB and ACV wrote the manuscript with edits from SN, ML, L-JS, SR, and PG. All authors contributed to the article and approved the submitted version.
