# Supplementary material for: β-D-XYLOSIDASE 4 modulates systemic immune signaling in Arabidopsis thaliana
Source: Front Plant Sci. 2023 Feb 2;13:1096800. doi: 10.3389/fpls.2022.1096800 (PMC9931724; doi:10.3389/fpls.2022.1096800)
Supplement: Supplementary file 1 [file DataSheet_1.pdf]

Supplementary Material

β-D-xylosidase 4 modulates systemic immune signaling in *Arabidopsis thaliana*

Kornelia Bauer, Shahran Nayem, Martin Lehmann, Marion Wenig, Lin-Jie Shu, Stefanie Ranf, Peter Geigenberger, A. Corina Vlot*

*** Correspondence:** A. Corina Vlot: corina.vlot@uni-bayreuth.de

Supplementary Table 1 Primers used to confirm T-DNA insertions in genomic DNA.

Name	Sequence 5′ → 3′	Description
**LBb1.3**	ATTTTGCCGATTTCGGAAC	LB primer for SALK mutants, T-DNA
**BXL4-3 LP**	ACTCCATCAAAACAAACGCAC	LP primer for BXL4-3, SALK_048903
**BXL4-3 RP**	GAATACCGACTCGCTGATCTG	RP primer for BXL4-3, SALK_048903

Supplementary Table 2 Primers used for qRT-PCR analysis

Name and description	Sequence 5′ → 3′	T_m [°C]	Reference
***UBIQUITIN* forward**	AGATCCAGGACAAGGAGGTATTC	66	(Breitenbach et al., 2014)
***UBIQUITIN* reverse**	CGCAGGACCAAGTGAAGAGTAG	67	(Breitenbach et al., 2014)
***BXL4* forward**	TGAGACCCGATAAAGCAAGCG	67	
***BXL4* reverse**	CGAGACCGAGAGAAACGAGAC	67	
***PR1* forward**	CTACGCAGAACAACTAAGAGGCAAC	68	(Breitenbach et al., 2014)
***PR1* reverse**	TTGGCACATCCGAGTCTCACTG	69	(Breitenbach et al., 2014)
***PDF1.2* forward**	CCAAGTGGGACATGGTCAG	66	
***PDF1.2* reverse**	ACTTGTGTGCTGGGAAGACA	67	
***LLP1* forward**	TGAGTAAACAGCAGTTACGA	60	(Breitenbach et al., 2014)
***LLP1* reverse**	TGACGCCATCAGAAGCAGGA	69	(Breitenbach et al., 2014)
***FMO1* forward**	ATCCCTTTATCCGCTTCCTCAA	66	(Bauer et al., 2021)
***FMO1* reverse**	CTCTTCTGCGTGCCGTAGTTTC	68	(Bauer et al., 2021)

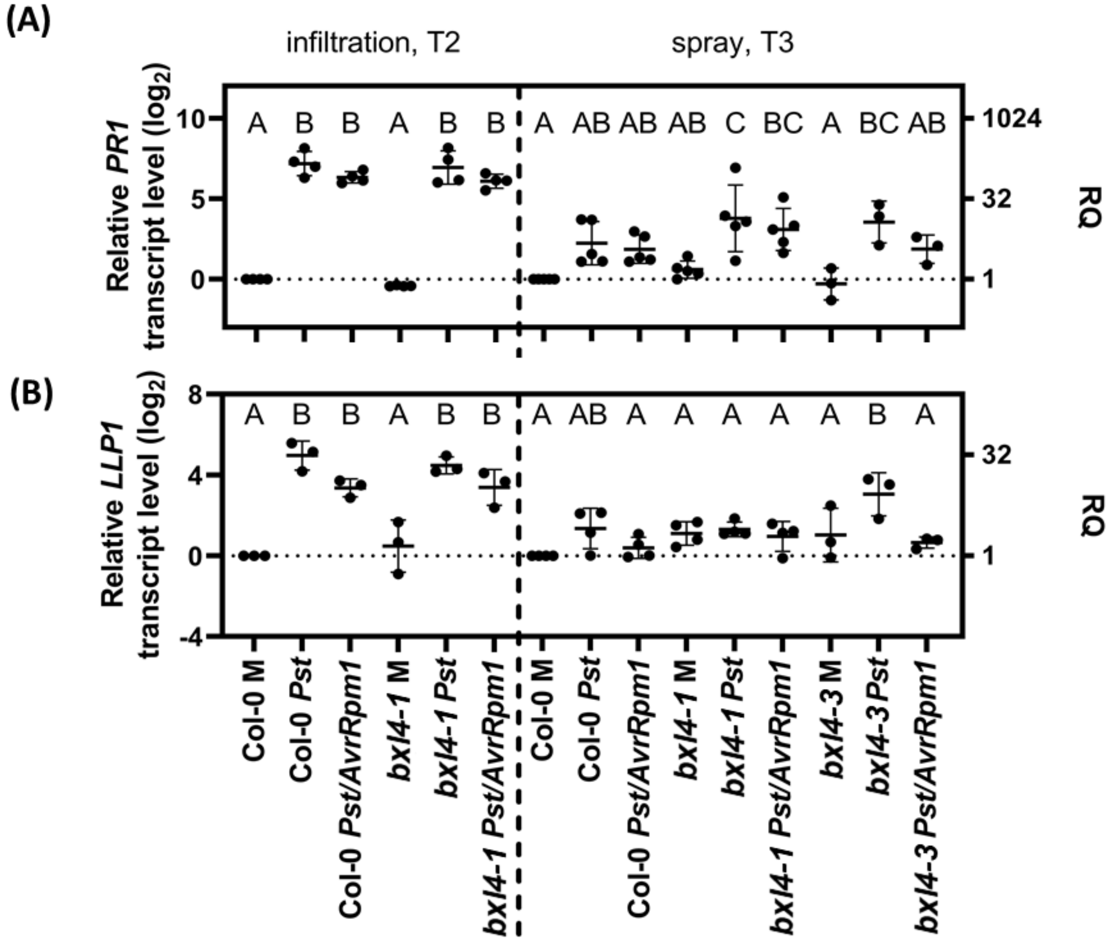

Supplementary Figure 1 Transcript accumulation of defense-associated genes in response to *Pst* and *Pst/AvrRpm1* is comparable in Col-0 and in *bxl4* mutant plants. Col-0 and *bxl4* plants were inoculated by syringe infiltration with 10^5 cfu/mL of *Pst* or *Pst/AvrRpm1* or by spray treatment with 10^8 cfu/mL of *Pst* or *Pst/AvrRpm1*. Transcript abundance of *PATHOGENESIS-RELATED PROTEIN 1* (*PR1*; **A**) and *LEGUME LECTIN-LIKE PROTEIN 1* (*LLP1*; **B**) in inoculated leaves was determined by qRT-PCR at 2 (T2) or 3 days (T3) post-inoculation. Transcript accumulation was normalized to that of *UBIQUITIN* and is shown relative to the normalized transcript levels in the appropriate Col-0 mock (M) controls. Black dots represent biologically independent data points and horizontal lines represent mean values ± SD from three to five biologically independent replicates. The letters above the scatter dot plots indicate statistically significant differences (one-way ANOVA and Tukey's test, P=<0.05, for (*PR1*: infiltration T2): n=4, F(5, 18)=155.3; for (*PR1*: spray T3): n=3-5, F(8, 30)=6.589; for (*LLP1*: infiltration T2): n=3, F(5, 12)=22.96; for (*LLP1*: spray T3): n=3-4, F(8, 24)=4.504).

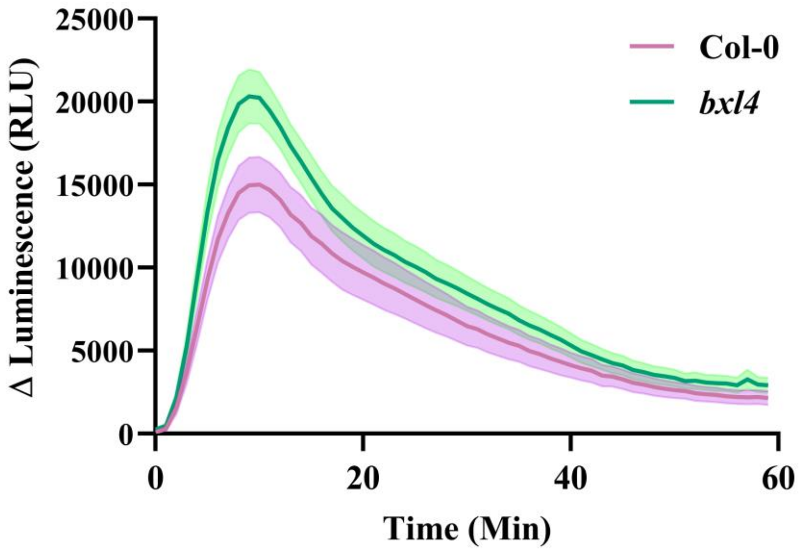

Supplementary Figure 2 Flg22-induced ROS burst is enhanced in *bxl4-1* mutant plants. Kinetics of ROS production in *A. thaliana* wt (Col-0) and *bxl4-1* (*bxl4*) leaf discs upon elicitation with 100 nM flg22. Data are depicted after normalization to average ROS levels 5 min before elicitor application and subtraction of water controls that were included for each genotype on the same plate. Data (relative light units, RLU) are pooled from three biologically independent experiments and are shown as mean ± SEM (number of leaf discs per genotype: n = 28 for flg22; n = 20 for water).

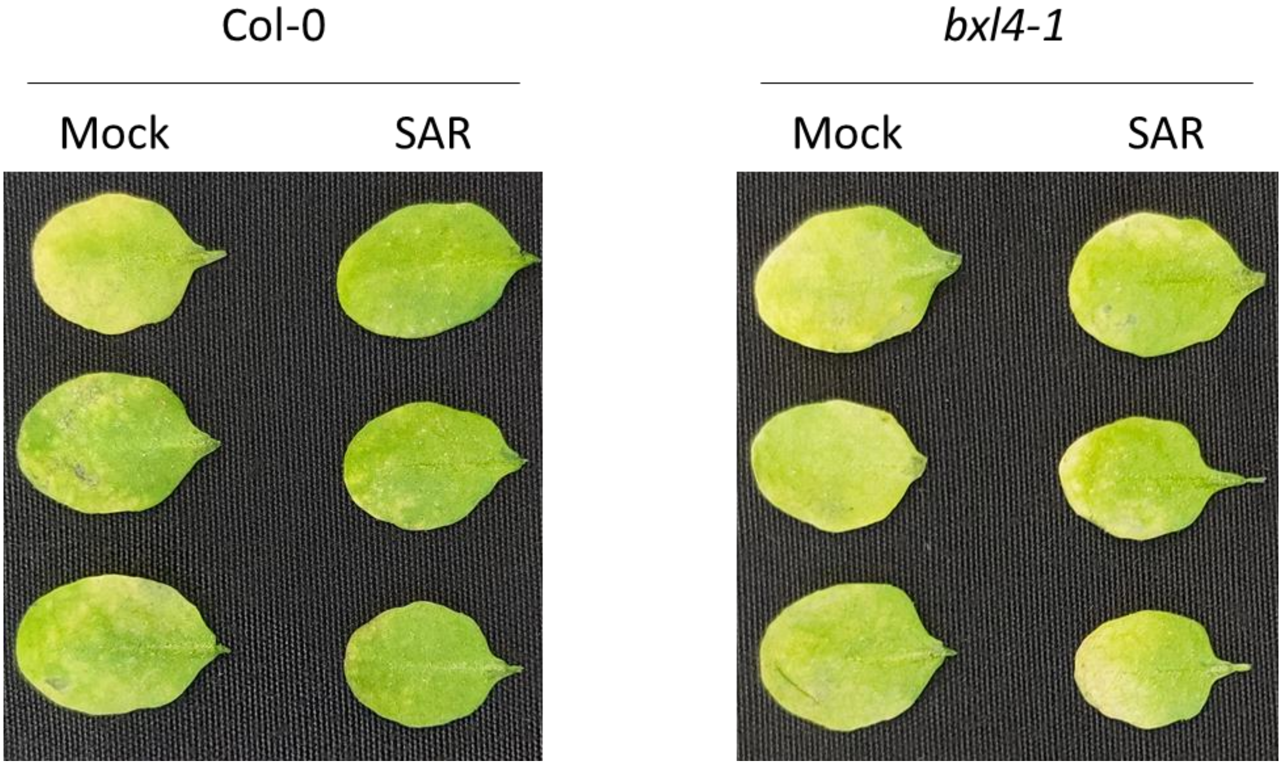

Supplementary Figure 3 *Pst* infection-associated symptoms after challenge-inoculation of leaves of SAR-induced and mock-treated wt and *bxl4-1* plants. 4-to-5-week-old plants were infiltrated in their first two true leaves with *Pst/AvrRpm1* (SAR) or a corresponding mock solution (Mock). Three days later, the systemic leaves were challenge-inoculated with *Pst*. The *Pst*-inoculated leaves were detached and images taken 4 days post-inoculation. Images from a representative experiment are shown.

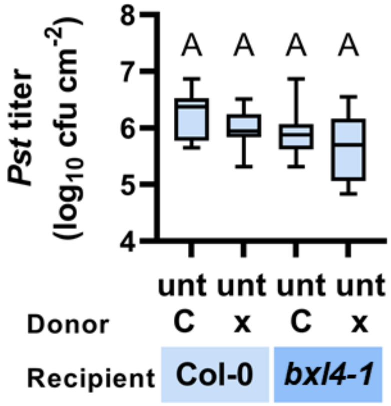

Supplementary Figure 4 *BXL4*-inducible molecules might accumulate in PetEx after inoculation or wounding. Leaves from untreated (unt) Col-0 (C) and *bxl4-1* (x) donor plants were cut at the middle of the plant rosette in order to retain their petioles and used to collect petiole exudates (PetEx). Subsequently, these exudates were syringe-infiltrated into naïve Col-0 and *bxl4-1* recipient plants. One day later, the inoculated recipient leaves were challenged with 10^5 cfu/mL of *Pst*. Bacterial titers in challenge-inoculated recipient plants were monitored at 4 dpi. Box plots represent average *Pst* titers in Col-0 and *bxl4-1* recipient plants from four biologically independent experiments ± min and max values. Letters above the box plots indicate statistically significant differences for means (one-way ANOVA and Tukey's test for P=<0.05, F(3, 66)=4.705, Col-0 + Col-0 unt n=20, Col-0 + *bxl4-1* unt n=19, *bxl4-1* + Col-0 unt n=16, *bxl4-1* + *bxl4-1* unt n=15).

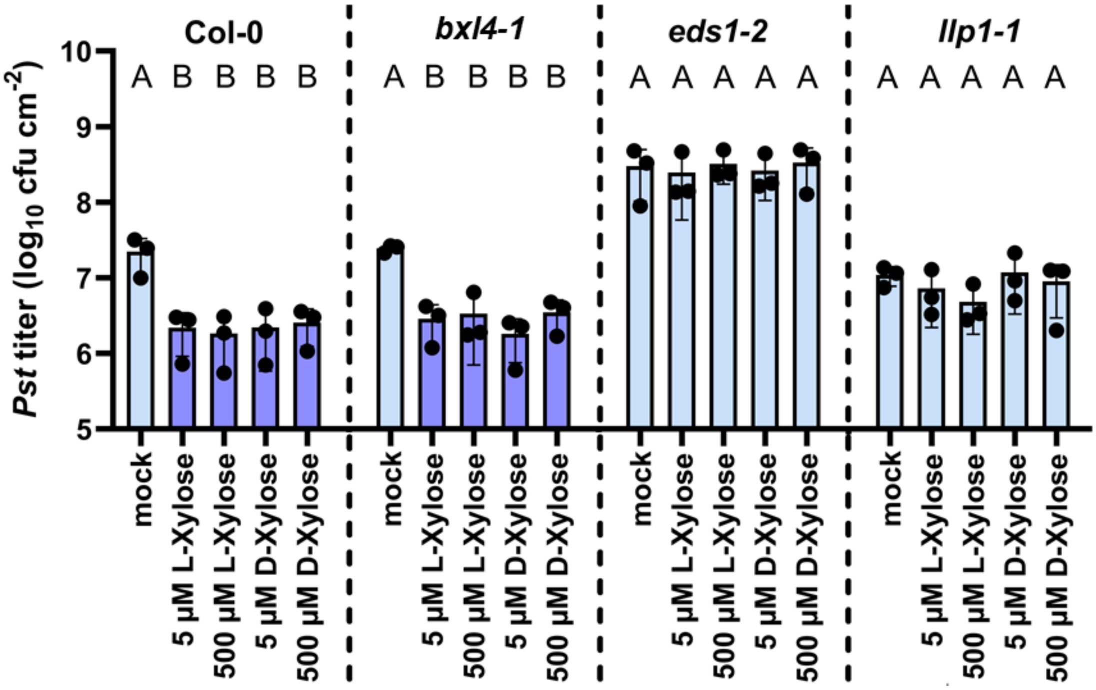

Supplementary Figure 5 Exogenous xylose induces *EDS1*- and *LLP1*-dependent systemic defense against *Pst*. Plants of the genotypes Col-0, *bxl4-1*, *eds1-2*, and *llp1-1* were inoculated with D- or L-xylose (dose as indicated below the panels), or with a corresponding mock treatment. Three days later, two distal leaves were inoculated with 10^5 cfu/mL of *Pst* and monitored for *in planta* bacterial titers 4 days after. Box plots represent average *Pst* titers of three biologically independent experiments ± min and max value. Different letters above box plots indicate statistically significant differences for means (one-way ANOVA and Tukey's test for P=<0.05, for (Col-0): n=3, F(3, 10)=12.66; for (*bxl4-1*): n=3, F(3, 10)= 18.02; for (*eds1-2*): n=3, F(3, 10)=1.06; for (*llp1-1*): n=3, F(3, 10)=0.896).

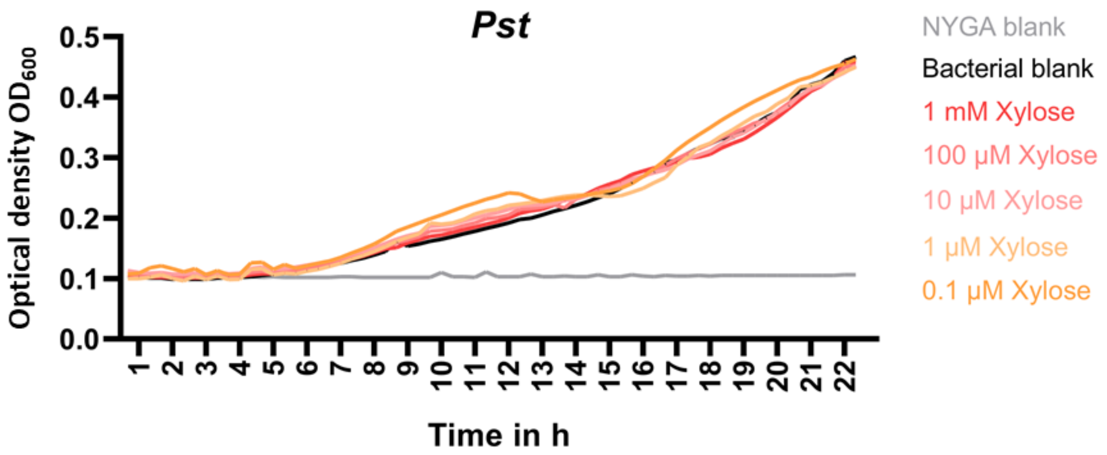

Supplementary Figure 6 Xylose does not affect the growth of *Pst* bacteria in liquid media. An initial inoculum with 10^7 cfu/mL *Pst* bacteria was grown for 22 h in liquid NYGA medium (bacterial blank) or in NYGA medium supplemented with a specific D-/L-xylose dose (as indicated). NYGA medium without bacteria was included as the NYGA blank control. The optical density at 600 nm (OD_{600}) of the suspensions was monitored every 20 seconds as a measure of bacterial density/growth. The OD_{600} mean values (n=4) were plotted.

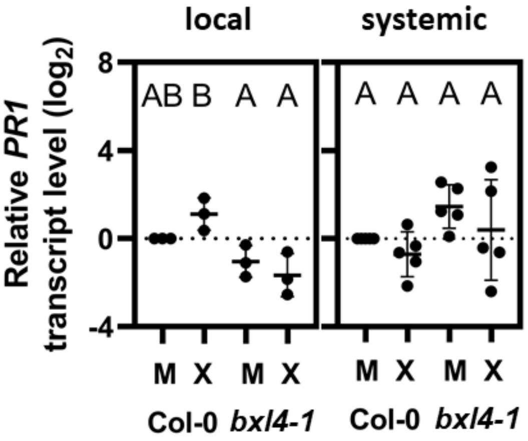

Supplementary Figure 7 Exogenous xylose does not significantly affect *PR1* transcript accumulation. Plants of the genotypes Col-0 and *bxl4-1* were inoculated with a dose of 10 µM D-/L-xylose (X) or with a corresponding mock (M) treatment. Three days later, *PR1* transcript accumulation was determined in the treated and in systemic leaves. Gene transcript accumulation was analyzed by qRT-PCR, normalized to that of *UBIQUITIN,* and is shown relative to the normalized transcript levels of the appropriate Col-0 mock controls. Black dots represent three to five biologically independent data points, and lines indicate the respective mean values ± SD. The letters above the scatter dot plots indicate statistically significant differences (one-way ANOVA and Tukey's test, P=<0.05, for (local): n=3, F(3, 8)=8.812; for (systemic): n=5, F(5, 15)=2.713).
